# Generative AI in hepatology: Transforming multimodal patient-generated data into actionable insights

**DOI:** 10.1097/HC9.0000000000000683

**Published:** 2025-07-14

**Authors:** Mason Lai, Irene Y. Chen, Jennifer C. Lai, Jin Ge

**Affiliations:** 1 Department of Medicine, University of California, San Francisco, California, USA; 2 UC Berkeley and UCSF Joint Program in Computational Precision Health, Berkeley, California, USA; 3 Department of Medicine, Division of Gastroenterology and Hepatology, University of California, San Francisco, California, USA; 4 Department of Medicine, Division of Clinical Informatics and Digital Transformation, University of California, San Francisco, California, USA

**Keywords:** artificial intelligence, cirrhosis, generative artificial intelligence, home monitoring, patient-generated data

## Abstract

Cirrhosis care is inherently complex, marked by a high risk of acute decompensation and significant morbidity and mortality. Traditional episodic care models provide static snapshots of a patient’s condition, limiting the ability to address dynamic changes in clinical status. Emerging at-home monitoring technologies and wearable devices present numerous opportunities to generate continuous clinical data, but the integration of this data into clinical workflows remains challenging. Large language models (LLMs) and generative AI (GenAI) technologies offer innovative solutions by enabling the acquisition, summarization, and actionable analysis of multimodal data generated by patients. This review explores the application of home-monitoring technologies for key complications of cirrhosis, including HE, ascites, and frailty. GenAI will enable the integration of these home-based data with canonical clinical data acquisition. We discuss the role of GenAI technologies and LLMs in processing multimodal data, supporting clinical decision-making, and creating autonomous artificial intelligence (AI) agents capable of triaging and summarizing patient data. In addition, we offer perspectives on the clinical evaluation of these emerging technologies. Finally, we close with a “not-so-distant” vision of GenAI-enabled at-home cirrhosis care.

## Graphical abstract

**Figure:**
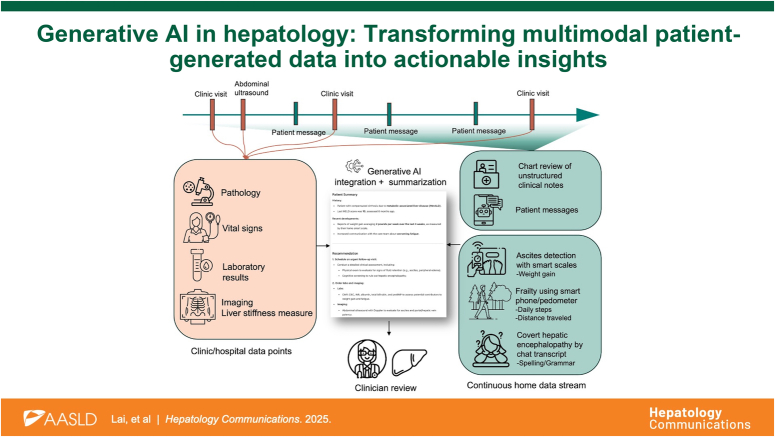

## INTRODUCTION

Caring for patients with cirrhosis is challenging due to its multifaceted nature, risk of acute complications, and overall high burden of morbidity and mortality. Timely identification of patients at high risk of decompensation, as well as care delivery systems that can reliably target resources to these patients at scale, remain an unmet need. Traditional care models have centered around scheduled clinic visits, either in-person, via video, or via telephone. These visits present a static snapshot-in-time of the clinical status of a patient with cirrhosis—one whose condition is often dynamic and could quickly decompensate. In contrast, at-home monitoring technologies and wearable devices can generate up-to-date patient data, opening a continuous window into the health of a patient outside of the clinical setting. To this end, there has been considerable effort spent developing systems and technologies that track various aspects of liver disease at home, but thus far, these interventions have not been widely implemented, thereby limiting their ability to affect patient outcomes. A substantial portion of these challenges is the technical integration of ever-increasing volumes and varieties of such data into existing clinical workflows.^1^^,^^2^

Large language models (LLMs) are a subset of deep learning artificial intelligence (AI) models trained on large quantities of text, thereby allowing them to mimic human language by predicting the next word in a sequence. As LLMs can not only predict but also generate new text, LLMs and related technology have been referred to as generative AI (GenAI), a new branch of AI that is now considered to be distinct from traditional machine learning and AI. Besides generating human-like text, GenAI models have demonstrated other, more sophisticated multimodal capabilities, such as image processing, code generation, and sentiment analysis.^3^ Due to these capabilities, GenAI technologies have the potential to enable the acquisition, filtering, and processing of high volumes of patient-generated data to allow it to be actionable for clinicians. Additionally, LLMs are able to handle complex reasoning and unstructured data, distinguishing GenAI from older ML methods. LLMs continue to show new capabilities as computational capacity scales, and architecture and training methods improve.^4^ Summarization and processing of patient-generated data using existing technologies can enable easier integration into clinical workflows, effectively serving as a link between unwieldy data streams and the clinical care team.

In this review, we will highlight examples of existing technologies that may be used to generate patient data at home, organized by specific complications of advanced liver disease. We will then discuss emerging applications of LLMs and GenAI technologies in relation to patient-generated data, and finally conclude by describing the potential evaluation of GenAI in the clinical setting.

## PATIENT DATA GENERATION BY DECOMPENSATION EVENT

### Hepatic encephalopathy

HE is a clinical diagnosis, identified by clinicians using patient-reported symptoms and exam findings. In its early stages, HE is termed covert hepatic encephalopathy (CHE). Patients with CHE show minimal clinical signs or symptoms. Up to 30%–85% of patients with cirrhosis have CHE.^5^ Diagnosis of CHE can be time-consuming, involving the administration of neuropsychological or psychometric tests, with the current gold standard being the psychometric hepatic encephalopathy score (PHES). Currently, the Stroop/EncephalApp is the only test that has been validated for home use for detection of CHE.^6^^,^^7^

In patients with dementia, audio transcripts can be broken down into various components that estimate syntactical and lexical features. These can, in turn, be analyzed by more traditional machine learning or language models to effectively distinguish healthy controls from patients with probable Alzheimer disease.^8^^,^^9^ Similarly, analysis of audio recordings by speech-language pathologists can differentiate between patients with overt HE, minimal HE, and healthy controls.^10^ The use of a remote data transmission app for caregivers of patients with cirrhosis was effective in reducing HE-related admissions by tracking medication adherence, sodium intake, and cognitive assessments.^11^ Sleep–wake disturbance is an early feature of HE, manifesting even in CHE. Commercial wearable fitness technology is able to track sleep quality, which, when combined with traditional serum biomarkers, can identify patients with CHE.^12^ HE is currently challenging and time-consuming to diagnose, particularly in its early stages. Wearable technology, voice recordings, and written conversation transcripts represent avenues through which CHE may be identified, without requiring the administration of lengthy psychometric testing.

### Ascites

Ascites is a common initial decompensating event, with up to 5%–10% of patients with compensated cirrhosis developing ascites each year.^13^ The development of ascites places patients at risk for further complications, including acute kidney injury, hepatorenal syndrome, infection, and hyponatremia. Furthermore, while diuretics are a mainstay of ascites management, overuse may lead to complications such as acute kidney injury. Conversely, diuretic underuse may lead to the accumulation of ascites requiring paracentesis or hospitalization.^14^ Despite best efforts to adjust diuretics based on daily weights, titration is challenging outside of the clinic setting. Smart scales are scales with the ability to automatically transmit data to a centralized system. The use of smart scales may provide a window into a patient’s status in the home, allowing for better-informed titration of diuretics. The use of smart scales has been best studied in the cardiology and heart failure literature. Prior meta-analyses have shown that remote patient monitoring (a combination of telehealth, wearables, and weight transmission) is associated with reduced mortality and hospitalization in heart failure.^15^ Randomized controlled trials using scales with automatic transmission of weights and a clinician alert workflow have shown improvement in quality of life, but have not shown reduction in rehospitalization or mortality, perhaps in part due to low rates of adherence to telemonitoring.^16^^,^^17^

### Frailty

Frailty has become recognized as a reliable marker of outcomes in hepatology, particularly in the liver transplant setting.^18^ In its original form, the Liver Frailty Index (LFI) requires in-person testing of grip strength, time to perform 5 chair stands, and seconds the participant can hold 3 different positions. With the onset of the COVID-19 pandemic and the subsequent rise in telemedicine, the TeleFi index, which consists of a functional capacity survey and optional visually assessed chair stands, was developed, which allows a clinician to assess frailty virtually, with good concordance with the original LFI.^19^ Repeated remote measurements can allow for continuous assessments of frailty, which ultimately could be used to proactively identify patients who would benefit from rehabilitation or physical therapy interventions.

Data from commercial smartwatches has been shown to correlate with gold standard measures of functional capacity (6-min walk test) for patients undergoing elective surgery.^20^ GPS-enabled smartphones have been used to generate measures of geographical territory, such as area and perimeter, which in turn correlate strongly with functional status and differentiate between healthy controls and patients with Alzheimer disease.^21^ Global Positioning System (GPS)-based mobility measures from smartphones have also been shown to correlate well with patient-reported outcomes (PROs) in the spine surgery setting,^22^ and in the breast cancer space—distinguishing patients recovering from breast-conserving surgery and mastectomy.^23^ Data from wearables could be used in conjunction with moments in time measurements, such as LFI, to give a more comprehensive sense of functional baseline at home and away from home.

### Patient symptoms and reported outcomes

As LLMs have grown increasingly sophisticated, they are now able to converse with patients, provide tailored education on their disease, and generate PROs. A variety of AI-focused techniques have opened the door to customizing and operationalizing these models for specific diseases and context-specific use cases, such as within hepatology.

LiVersa, a liver disease-specific LLM created by retrieval augmented generation on 30 AASLD guidance documents, was developed using a HIPAA-compliant version of GPT-4. Its outputs were rated by hepatologists as more accurate compared to ChatGPT 4 and LLaMA 2.0.^24^ While LiVersa was primarily intended to be a clinician-focused application of an LLM-enabled Chatbot, there is also potential for patient-facing applications. Outside of hepatology, LLMs have been shown to be capable of generating follow-up questions to patient messages, with responses that were rated similarly to physician-generated responses.^25^^,^^26^ Similarly, LLMs have also been shown to be able to generate responses, including recommendations and next steps, to patient messages in the primary care setting, that were rated accurate and responsive.^27^

PROs are validated instruments that are gaining interest as a quality metric within hepatology, given their high concordance with clinical outcomes.^28^^–^^30^ A study of PROMIS-29 within hepatology showed associations with hospitalization.^31^ While the strength of PROs lies in their validity, their administration can be time-consuming—the flexibility of LLMs offers an alternate avenue to access patient-centered outcomes. Chatbots are capable of administering PRO instruments in a conversational format,^32^ which can then be summarized, processed, and analyzed for clinician review. This, in turn, could focus on in-person or virtual clinical visits and potentially allow for timely interventions. This is an area of further exploration as indicated by ongoing studies in using natural language processing to extract PROs from unstructured text, although the use of LLMs for this purpose remains rare.^33^

## INTEGRATION AND CLINICAL DECISION SUPPORT

### Integration of multimodal data

The current volume and complexity of healthcare data present significant challenges for clinical management, particularly in hepatology, where disease progression and management are complex and affected by a wide range of variables. GenAI technologies LLMs offer a notable advantage over traditional machine learning systems due to their ability to incorporate multimodal data formats, including unstructured and imaging data. This flexibility could streamline the integration of patient-generated data from devices at home, such as wearable devices, without the need for manufacturer-specific electronic health record (EHR) integration—thereby decreasing costs for implementation of these technologies.

**FIGURE 1:**
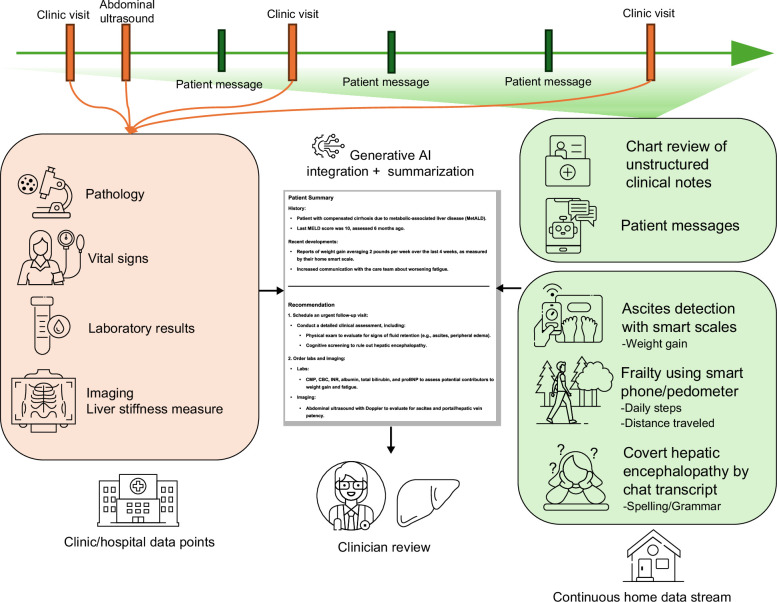
Future state of GenAI data integration and applications in hepatology.

The most salient advantage of GenAI for data integration is summarization for clinical interpretation, thereby saving clinicians’ time and cognitive effort. For example, patient-generated data using GPS signals, smart scales, or transcripts that indicate risk for CHE can be summarized by GenAI and sent as a message to the clinician’s inbox, providing an effective “digital phenotype,” available for review prior to a clinic visit (Figure 1). While clinical decision support is already embedded in modern EHR systems, these are largely limited to the use of structured data and preset rule-based algorithms. Such integration is likely to be labor intensive and costly, with estimates for implementing new clinical decision support systems in EHR ranging from $48,669 to $201,500.^34^^–^^36^ The high upfront cost and difficulty of implementing clinical decision support is multifactorial and is partly driven by EHR heterogeneity and difficulty integrating data that is not streamlined or compatible with EHR.^37^ In contrast, GenAI can incorporate large volumes of both structured and unstructured data to identify trends or predict risk—such as impending patient decompensation—for clinician review. An example of the use of unstructured data is sentiment analysis, the process of extracting a quantity representing an opinion or thought of a given text, when applied to free-text nursing notes, can predict rehospitalization.^38^^,^^39^

### AI agents

GenAI technologies also enable the development of “AI agents,” which could operate autonomously or with minimal human input—analyzing data, identifying patterns, making decisions, and taking specific actions within a limited space.^40^ These agents may represent the next step in the implementation of LLMs and GPTs by enabling LLMs to autonomously perform a limited set of actions and are already being deployed in customer-facing applications in business. A hepatology AI agent may work in conjunction with an existing home-based model of cirrhosis, such as CirrhoCare,^41^ wherein patients used a smartphone app to transmit data to their hepatologist (weight, hemodynamics, symptoms), with 2-way communication between clinician and patient. The hepatology AI agent would assume the intermediary role of summarizing data, identifying trends, and providing simple guidance to patients, flagging the clinician for review when appropriate. Triaging patients remains a high-stakes clinical decision, particularly within cirrhosis care, given its complex nature and the often-rapid tempo of decompensation.

In this theoretical application of a hepatology AI agent, GenAI technologies and LLMs may serve as a first-pass filter to offload the triaging clinician, but ultimately, this decision should remain under clinician supervision. In nephrology, pilot studies have shown that GPTs are able to appropriately triage 93% of simulated patient messages.^42^ GPTs have already shown promise in the ability to triage in the emergency department setting, with the ability to accurately identify higher-acuity patients between a pair of initial histories, highlighting their potential to be effective decision-support.^43^ Similarly, LLMs have previously been able to predict hospital admission based on nurse triage notes.^44^ Additionally, mixed methods studies have shown that patients have favorable opinions when interacting with GenAI^45^^,^^46^ although patients are currently uncomfortable with heavy reliance on GenAI.^47^ Within hepatology, these applications might focus on identifying patients at risk of developing impending spontaneous bacterial peritonitis, variceal bleeding, or exacerbation of HE. For instance, the hepatology AI agent could receive data from a patient’s home devices indicating rapid weight gain and reduction in travel distance from home, interpret these data as high risk for spontaneous bacterial peritonitis and/or acute kidney injury, and automatically flag the clinician to review the patient’s chart, schedule an urgent visit in clinic, message the patient and gather symptoms in parallel. Accuracy, usability, and impact on clinical workflow will require context-specific evaluation to determine if an AI agent would benefit clinician workflow, patient experience, and improve outcomes.

## GenAI EVALUATION AND VALIDATION

**FIGURE 2:**
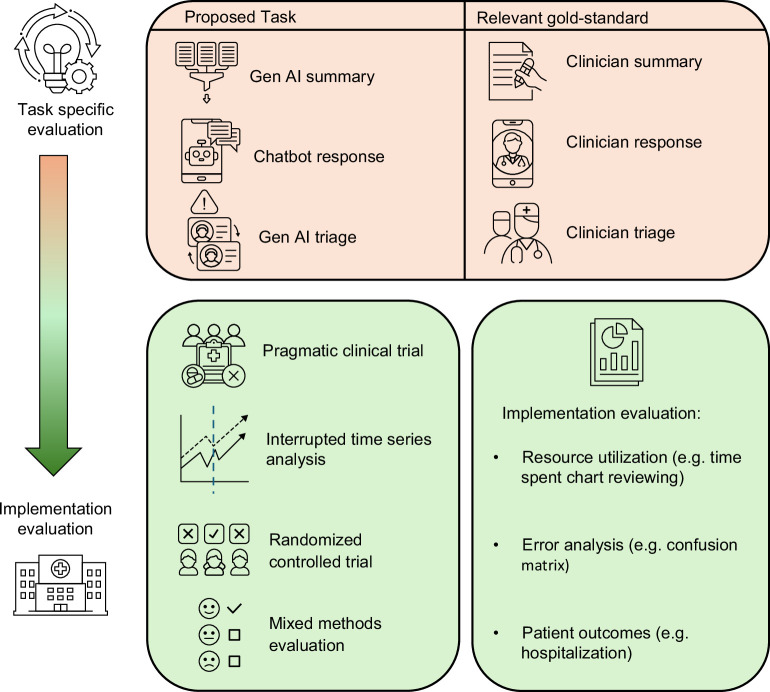
Evaluation of GenAI in hepatology. Abbreviation: GenAI, generative AI.

As with any new clinical technology, GenAI applications in hepatology will require validation before it is ready for large-scale clinical implementation. Given the nascent nature of GenAI in healthcare, formal standards for validation do not yet exist. We propose dividing the evaluation process into 2 stages: (1) Task-specific, where GenAI is evaluated for the performance of a specific task in a research setting; and (2) Implementation, where GenAI is evaluated after implementation in a clinical setting. We anticipate that validation will often be sequential, with initial evaluation being task-specific to determine if GenAI performs sufficiently, followed by implementation evaluation to determine its efficacy in the clinical realm (Figure 2).

Within the task-specific group, validation will likely take on different forms depending on the application of AI, but will ultimately require testing in comparison to the relevant gold standard. For example, in the domain of patient communication, evaluations of GenAI-produced responses (eg, to simulated patient messages) have been conducted by rating the AI-generated responses to those created by clinicians based on several criteria.^25^^,^^27^ In the domain of medical decision-making, the ability to triage emergency department and outpatients has been evaluated by comparing decisions made by GenAI against clinician triage decisions.^42^^,^^43^ In another example, the ability of AI to identify language patterns in patients with CHE could be performed by comparing the AI assessment against PHES testing.

As GenAI applications and use-cases expand, assessment of their abilities to improve patient outcomes in the clinical setting is likely the next step, shifting evaluation methods toward those used in implementation science. This involves studying not only patient outcomes (both hard outcomes and PROs), but process metrics such as adoption, decision change rates, and time spent on tasks like after-hours charting.^48^ Specific techniques that could be employed include interrupted time series analyses, where the projected trend of an outcome is compared against the observed trend following the implementation of an intervention.^26^ Pragmatic clinical trials aim to test the efficacy of an intervention in real-world settings, rather than conventional, highly controlled clinical trial settings, thereby increasing their generalizability and narrowing the gap from research to clinical practice.^49^

GenAI-enabled clinical decision support or AI agents could be evaluated by pragmatic clinical trials and serve to evaluate their ability to improve patient care. For instance, one such evaluation could be of rates of hospitalization among patients in EHR-implemented AI-assisted care models that summarize and triage patient messages, compared to the standard of care. Additionally, GenAI implementations would benefit from iterative and continuous evaluations after deployment to ward against potential harms posed by dataset shift and model biases. Of note, implementation of GenAI in clinical decision making will require a high level of FDA regulation and oversight as a medical device, given the risk posed to patient safety if the LLM does not function as intended.^50^ Evaluation of GenAI clinical decision support will have to follow existing established frameworks, such as the Practical, Robust, Implementation, and Sustainability Model (PRISM) framework.^48^^,^^51^

## CONSIDERATIONS FOR GenAI IMPLEMENTATION IN CLINICAL PRACTICE

### Bias

The safety and dangers of GenAI remain a topic of active discussion, and for good reason—as the role of AI in healthcare continues to evolve, new healthcare disparities may arise.^52^ Data used to train AI models must be chosen carefully, as these models can and will learn biases embedded within the training data. Rigorous review and active efforts to identify and combat these biases will become increasingly important as GenAI is implemented into the clinical workflow.

### Privacy and data security

Additionally, concerns around privacy and security of data used by GenAI are also warranted. Care must be taken around data transmission, storage, and compliance with privacy laws and regulations. Finally, the accuracy of these models is context and application-dependent—what is accurate for one group (eg, clinician) may not be accurate for another (eg, patient). Evaluation of GenAI will require an assessment that is tailored for specific use cases and target audiences.

### Cost

To the best of our knowledge, the cost of implementation of GenAI into an existing clinical system remains unknown. Implementation cost will certainly vary by system and by the scope and task assigned to GenAI. Additionally, the ongoing costs (ie, cost per token) of GenAI must be taken into consideration, although we expect that usage will become cheaper over time as computational capacity scales and more efficient methods are developed.^53^

## FUTURE DIRECTIONS

As liver disease care becomes increasingly digitized, the volume and variety of healthcare data will continue to grow.^1^ Transmission, consolidation, and application of this data represent an opportunity to gain insight into a patient’s continuous state in the community rather than the static snapshot afforded by traditional clinic models. Given the technological development in the last few years in GenAI, we are enthusiastic about reimagining clinical care for patients with chronic liver diseases.

A theoretical patient journey in the not-so-distant future (with home-monitoring technology and GenAI-augmented care) may look like the following for a patient, Joan, who has decompensated cirrhosis complicated by medically managed ascites and HE:

- Joan’s smartphone functions as a GPS/pedometer and notes that over the last week, she has only left her home once and is taking 30% fewer steps per day than her baseline. This trend is transmitted to a remote monitoring system, prompting a notification to check her weight and start a chat with a specialized hepatology chatbot.
- The scale weight is 5 pounds heavier than Joan’s weight one week ago. This data is transmitted to a remote monitoring system, triggering a notification to take an additional 20 mg of furosemide.
- Joan then initiates a chat with the specialized hepatology chatbot. The chatbot asks her questions about medication adherence, symptoms concerning infection, and confusion.
- Joan reports that other than generalized fatigue, there has not been any change in her routine or new symptoms. However, during the chatbot interaction, her spelling and grammar are abnormal, triggering an alert for potential encephalopathy. The specialized chatbot directs her to take an additional dose of her lactulose, and she is automatically scheduled for a call with a triage nurse that afternoon.
- Joan reports to the triage nurse that she has been using the restroom frequently following the additional furosemide, and she sounds clear and alert. The triage nurse determines that while she does not need to present to the emergency department, she would benefit from a video visit with her hepatologist to adjust her medications and schedule her for a video visit in 2 days.
- During the video visit, the hepatologist orders routine blood work and liver function tests, increases her furosemide and spironolactone doses, and starts her on rifaximin.
- One week after Joan’s video visit, her phone automatically prompts her for a scheduled check-in with a chatbot—during this short chat, Joan’s spelling and grammar are clear, and she reports her fatigue has resolved.
- Remote transmissions from her scale reveal that she is back at her baseline weight. The labs she obtained earlier in the week are stable from her baseline. These data are summarized and transmitted to Joan’s hepatologist’s inbox, who determines no further changes are needed and schedules a routine follow-up visit in 3 months.

As the above futuristic vision demonstrates—the opportunities for GenAI applications will only continue to grow as both home monitoring and GenAI mature. Sensible and rational integration of GenAI has the potential to enhance the delivery, accessibility, and quality of patient care to hepatology patients.
